# Anterior Traumatic Lumbosacral Dislocation: A Case Report

**DOI:** 10.7759/cureus.35518

**Published:** 2023-02-27

**Authors:** Oussama Lassioued, Walid Balti, Mehdi Bellil, Khaled Hadhri, Mondher Kooli, Mohamed Ben Salah

**Affiliations:** 1 Department of Orthopedic Surgery, Charles Nicolle Hospital, Tunis, TUN

**Keywords:** lumbar fusion, surgical treatment, fracture dislocation, traumatic injury, spondylolisthesis, lumbosacral spine

## Abstract

Traumatic dislocation of the lumbosacral joint is a rare and severe lesion usually caused by high-energy trauma. The literature on traumatic spondylolisthesis is limited, and most published papers are sporadic case reports.

By presenting the case of an anterior traumatic L5-S1 spondylolisthesis without neurological deficits caused by a 6-meter fall, we discuss the anatomopathological mechanism of this injury, clinical and radiological evaluation, and current management options. The patient was treated surgically with a combined posterior instrumented reduction and transforaminal interbody fusion. At the final follow-up after seven years, the radiological evaluation showed an unchanged spondylolisthesis reduction with reliable fusion healing. In addition, the patient had good functional results and resumed recreational activities and work.

Traumatic lumbosacral spondylolisthesis requires a careful and well-documented initial clinical and radiological assessment. Most authors advocate surgical treatment as the mainstay of management. However, the long-term prognosis remains unclear and unpredictable.

## Introduction

Traumatic dislocation of the lumbosacral joint is a rare and severe clinical entity, usually caused by high-energy trauma [1]. To date, the literature on traumatic spondylolisthesis is limited and sparse. Furthermore, most of the published papers are sporadic case reports, which explain the controversy surrounding this lesion's pathoanatomy, treatment, and prognosis. With this background, we present a case of a patient with traumatic L5-S1 anterolisthesis to discuss the mechanism of this injury, clinical and radiological evaluation, and recommended treatment options.

## Case presentation

A 41-year-old electrician with no medical history presented to the emergency department with excruciating low back pain after sustaining a fall from a height of 6 meters at work. On clinical examination, the patient was hemodynamically stable. He had a Glasgow Coma Scale score of 15/15 and was tender in the lumbosacral region without a step-like deformity or wounds. A detailed neurologic examination of the lower limbs revealed no sensory or motor deficits (American Spinal Injury Association (ASIA) score E). In addition, there was no perineal hypoesthesia or other features suggestive of bowel or bladder involvement (cauda equina syndrome).

**Figure 1:**
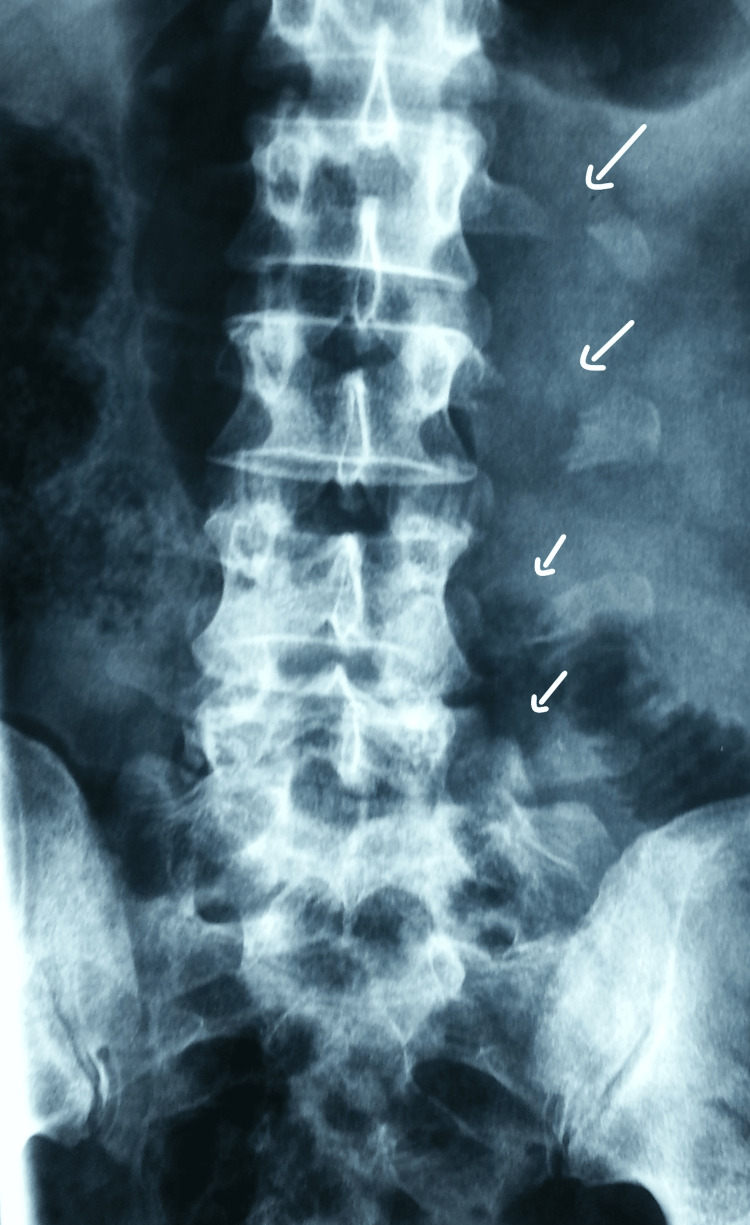
Anteroposterior X-ray revealing multiple transverse process fractures

**Figure 2:**
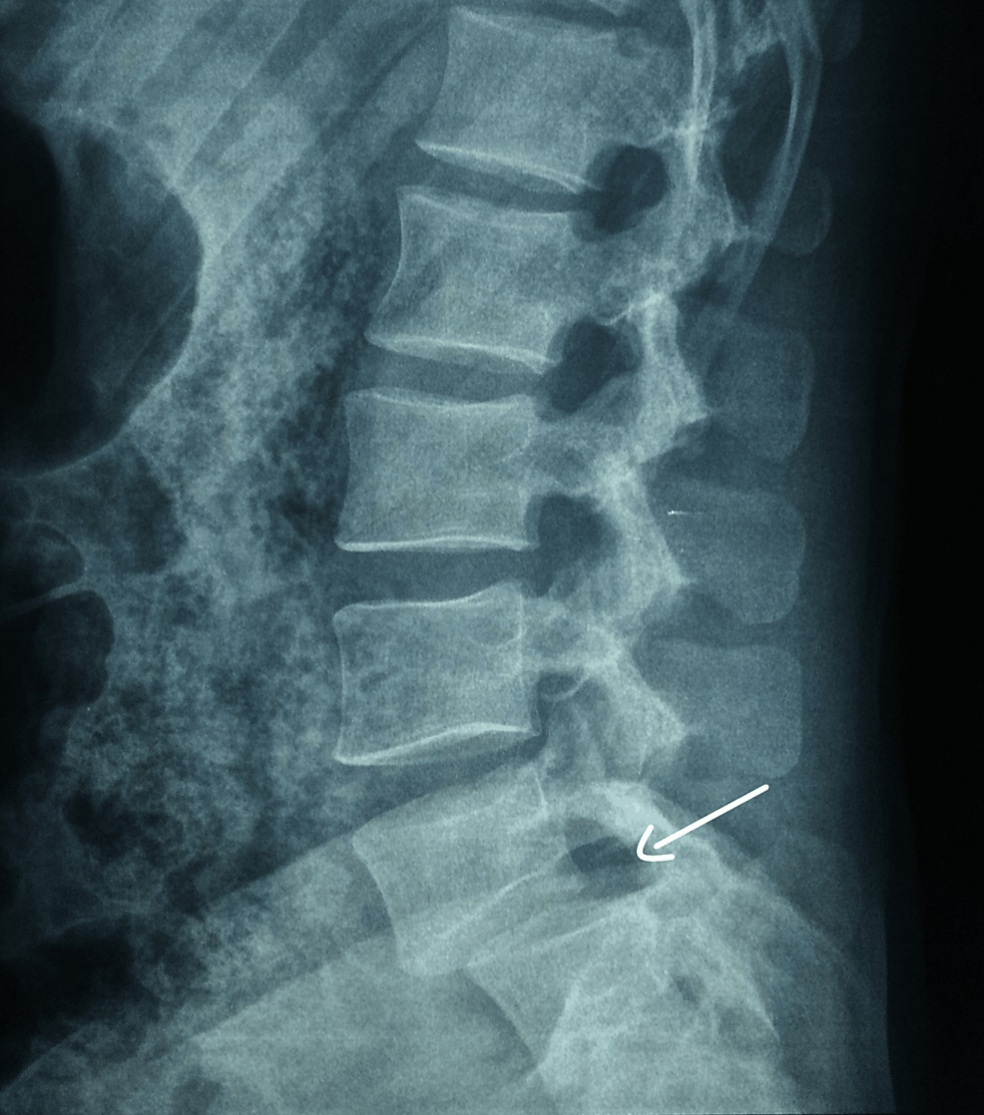
Lateral X-ray showing L5-S1 spondylolisthesis

Plain radiographs showed a multilevel fracture of the left lumbar transverse processes, which were laterally displaced (Figure 1), and grade II anterolisthesis of L5 on S1 without isthmic lysis (Figure 2).

**Figure 3:**
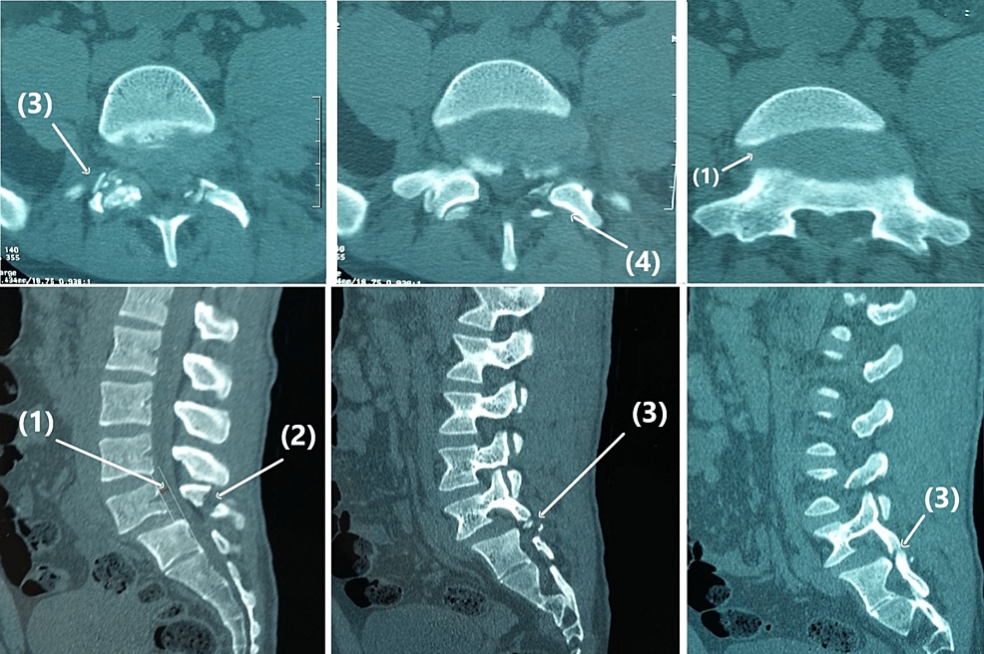
Axial and sagittal CT scans (1) L5-S1 spondylolisthesis. (2) L5 spinous process. (3) Facet dislocation and L5 inferior facet tip fracture. (4) Naked facets of S1.

Further evaluation by computed tomography (CT) of the lumbosacral spine revealed a bilateral comminuted fracture-dislocation of the inferior articular facets of the fifth lumbar vertebra with multiple fractures of the spinous process of L5 and the left transverse processes of the L1 to L5 vertebrae. The CT scan also showed moderate anterior displacement of L5 on S1 (>25%) and naked facets of S1 on the axial slides, confirming the diagnosis of acute anterior traumatic lumbosacral spondylolisthesis (Figure 3).

Magnetic resonance imaging (MRI) of the lumbosacral spine was not initiated. Ten days after the presentation, the patient was operated on in the prone position via a posterior approach. Surgical findings confirmed a bilateral facet dislocation, a fracture of the spinous process of L5, and a posterior ligamentous complex (PLC) injury. The procedure began with the removal of all fractured portions of L5 and bilateral foraminal decompression of the L5 nerve roots, which were undamaged. This step resulted in a spontaneous reduction of the fracture. Next, an L5-S1 discectomy was performed, followed by transforaminal interbody fusion with an oblique titanium cage, and an autologous corticocancellous bone graft from the decompression material was placed to ensure a higher fusion rate. A large cage was used to achieve adequate stability and restore optimal lordotic angulation.

**Figure 4:**
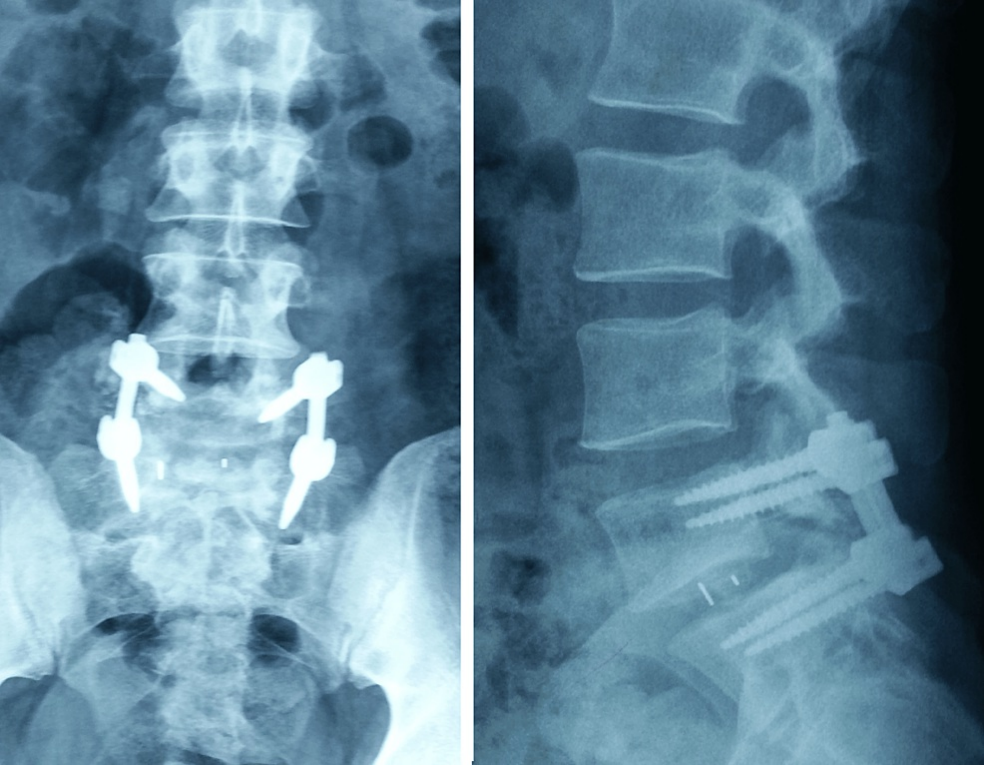
Postoperative radiographs demonstrating anatomic reduction and solid instrumented L5-S1 circumferential fusion

Posterior segmental reduction and stabilization were performed with L5-S1 transpedicular screws and pre-contoured rods. Finally, the transverse processes were decorticated, and a posterolateral autogenous bone graft was inserted (Figure 4).

**Figure 5:**
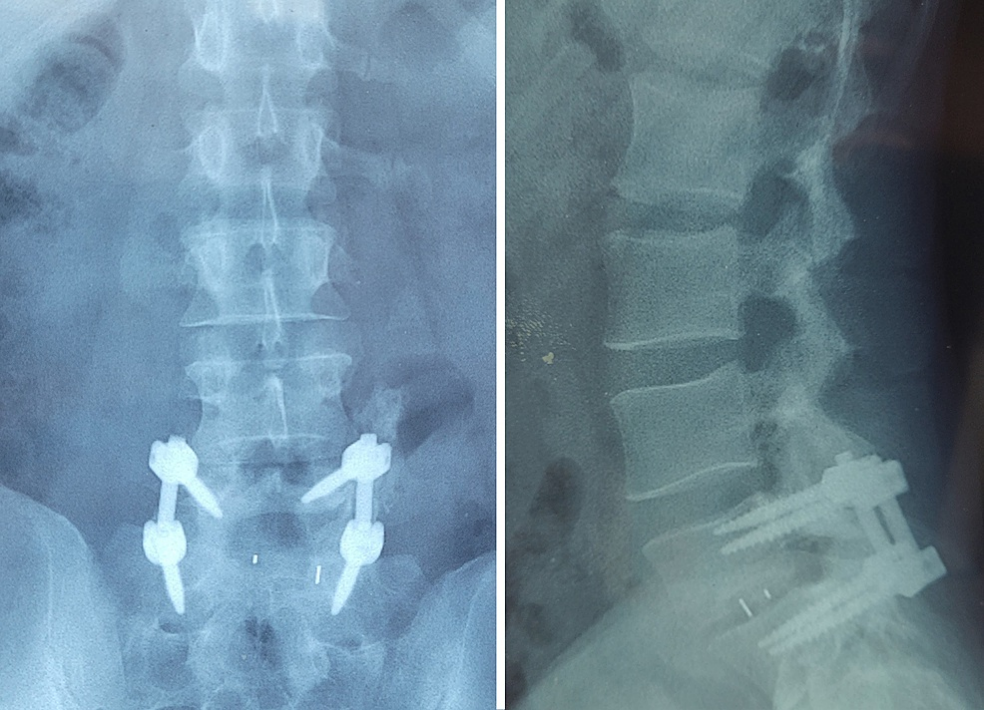
Seven-year postoperative radiographs demonstrating an anatomically aligned and preserved solid spinal fusion

The patient recovered without complications and was discharged four days after surgery with restrictions on forward bending and weight lifting. He was allowed to begin physiotherapy gradually. The patient returned to work six months after the injury and was reasonably satisfied. At the seven-year follow-up, he remained free of back pain and could resume his previous level of physical activity. Radiological evaluation showed unchanged spondylolisthesis reduction with reliable healing of the posterolateral and interbody bone grafts (Figure 5).

## Discussion

Wiltse classified lumbosacral spondylolisthesis into five types: isthmic, dysplastic, pathologic, degenerative, and traumatic [2]. The traumatic anterolisthesis type is a very uncommon lesion, first described by Watson-Jones in 1940 [1]. This entity is caused by a fracture of the posterior elements rather than the pars interarticularis, resulting in instability and vertebral slippage. Therefore, it should be distinguished from acute isthmic spondylolisthesis, in which a pars fracture occurs because of predisposed spondylolysis.

Several physiopathologic hypotheses about the mechanism have been suggested. However, most authors consider that the primary mechanism of anterior or anterolateral lumbosacral dislocation is hyperflexion associated with axial compression, usually due to seatbelt or lap-belt injuries without chest strapping during a motor vehicle accident [3].

There are also reports of other contributing forces, such as hyperextension with compression, rotation, direct traumatic vectors, and anterior translation, as possible mechanisms for traumatic anterolisthesis [4,5]. To date, no biomechanical study has elucidated the exact mechanism responsible for each variant of lumbosacral dislocation, and the cause of such injuries remains controversial [1,3]. The L5-S1 level is the most common site for traumatic spondylolisthesis. The transition from a sagittally oriented thoracolumbar facet joint to a coronally oriented lumbosacral facet joint explains the predisposition for disarticulation and anterolisthesis [4,6]. The anatomic inclination of the sacrum is also involved in the increased prevalence of traumatic spondylolisthesis at this level. Indeed, the greater the angle of the lumbosacral joint, the greater the translational forces applied [7,8].

In addition, the iliolumbar ligament complex plays a vital role in maintaining the stability of L5-S1. The rupture of the posterior ligament of this structure contributes to increasing the flexion arc of the lumbosacral joint by 77%. Therefore, fracture of the transverse process of L5 leads to disruption of the iliolumbar ligament and may explain traumatic listhesis [3].

Acute lumbosacral dislocation affects the young population after a violent trauma resulting in substantial spinal and ligamentous damage and strongly suggestive of the severity of associated injuries. Therefore, careful clinical assessment is necessary to identify life-threatening emergencies and to diagnose recurrent neurologic deficits such as lower extremity hypoesthesia, radiculopathy, bowel dysfunction, and urinary retention early [1].

In the literature, a wide range of neurological manifestations have been reported. Grivas et al. [9] found a 58% rate of neurologic damage in all lumbosacral fracture-dislocations. Aihara et al. [10] recorded a 68.4% rate of neurologic deficits in 57 cases, while Arandi et al. [11] found 89% of patients with neurologic impairment in complete lumbosacral dislocations. In rare cases, cauda equina syndrome may be present and should be permanently excluded. Otherwise, immediate surgical treatment should be performed [3,5].

Standard anteroposterior (AP) and lateral radiographs are the basis of diagnosis. The AP view shows transverse process fractures, which serve as a warning to the physician to look for traumatic spondylolisthesis. It is seen in 50-80% of cases and considered a "sentinel sign.''

AP radiographs may also show skewing of L5 on the sacrum, enlarging of the interpedicular distance, widening of the paravertebral soft tissue lines, and rotational deformation of the spinous processes. Lateral views may show increased interspinous distance, sharp kyphosis of L5 on S1, anterior subluxation at L5-S1, anterior narrowing of the height of the disk space, interrupted spinolaminar lines, or enhancement of lumbar lordosis [3,12,13].

CT is the best imaging modality for diagnosing traumatic spondylolisthesis, especially in complex lesions or subtle displacements. It provides excellent visualization of injuries to the posterior bony structures, including perched, locked, or fractured facets, as well as the "naked facet" sign in the axial plane and associated laminar, pedicular, or sacral fractures. Additionally, it permits measuring the diameter of the spinal canal. However, because CT is achieved in the supine position, anterolisthesis may be underestimated [8,12,14].

MRI is mandatory to critically assess the integrity of the disk contents, ligamentous structure injury, and nerve compression without delaying surgical treatment when cauda equina syndrome is clinically suspected [8].

**Figure 6:**
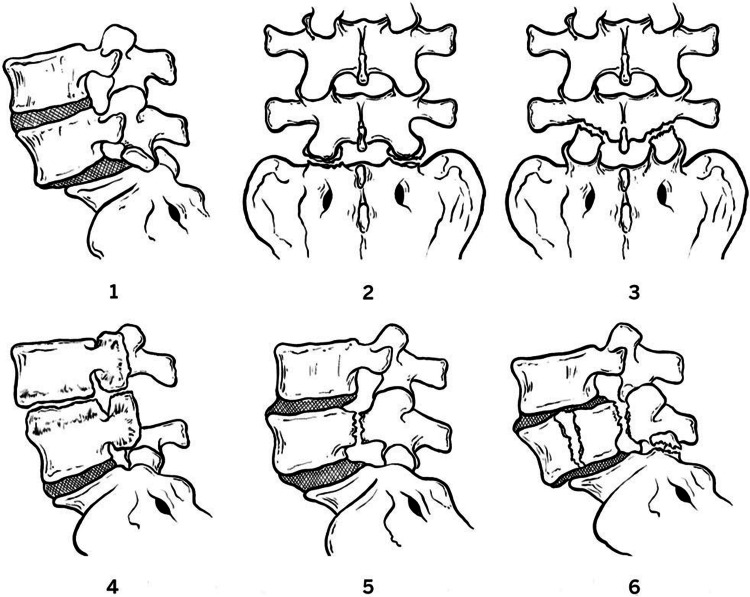
Traumatic lumbar spondylolisthesis classification according to Dimar Jr. II Low-grade: type 1: bilateral or unilateral facet dislocation; type 2: bilateral or unilateral facet fracture; and type 3: bilateral or unilateral pars fracture. High-grade: type 4: fusion mass fracture; type 5: bilateral pedicle fracture; and type 6: complex fracture-dislocation with vertebral body implication. Figure adapted from [4].

Numerous classification systems have been described in the literature. In 1998, Aihara et al. [10], after analyzing seven cases treated at their center and the existing literature (50 cases), introduced a new classification system (five types) based on the involvement of facets, the vertebral body, and posterior elements to guide surgical decisions. Vialle et al. proposed a classification system (three types with subdivisions) based on the mechanism of injury [8]. In 2018, Dimar Jr. II [4] recently classified traumatic spondylolisthesis according to the amount of involvement of the anatomical structure of the vertebra by reviewing 125 cases from the current literature (Figure 6).

The severity of the anterior displacement of a vertebra in spondylolisthesis is graded by the Meyerding grading system (Table 1) [15].

**Table 1:** The Meyerding classification grade Table adapted from [15].

Meyerding classification	Percentage of slip
Grade I	0-25
Grade II	25-50
Grade III	50-75
Grade IV	75-100
Grade V	>100 (spondyloptosis)

Different strategies and treatment modalities are proposed to manage lumbosacral dislocation. The main goals are preventing the progression of listhesis, neurologic deficits, and back pain. Although there are few reports of successful conservative treatment, failure or non-fusion with progressive spondylolisthesis and secondary neurological impairment were reported early in the literature [3,10,16-20].

Lumbopelvic dissociation is considered an unstable lesion regardless of the type of injury because it involves the three spinal columns and ruptures all stabilizing structures. Therefore, operative management is the mainstay of treatment [13,18,21]. The principle of surgery consists of decompression of the nerve roots, reduction of anterolisthesis, and stabilization of the spine [22].

In general, low-grade injuries (types 1-3) or those with less severe listhesis (less than 50%) require only posterior instrumentation and posterolateral fusion. The necessity of interbody fusion is controversial. Many authors have shown that in these types of lesions, there is no statistically significant difference in fusion rate, walking ability, pain, or neurological deficits between the groups with isolated posterolateral fusion and the groups with additional intercorporal fusion [3,4]. However, it is critical to rule out the neural canal and bilateral foraminal compression caused by neurotoxic bone or disk protrusion, particularly after reduction, which is required for spondylolisthesis grade 2 or higher [14,19,21]. A partial facetectomy can facilitate reduction, although intact apophyseal joints are preferred to prevent redislocation [1].

Otherwise, if the lesion involves the anterior column or pedicles (types 4-6) or if there is a high-grade slipped vertebra (>50%), posterior fixation and fusion, including additional levels proximal to L4 or distal to the pelvis (iliosacral or pelvic), are recommended to improve stability with possible anterior interbody fusion [21-23]. In the case of traumatic disk herniation, the treatment is modified: the disk should be removed for decompression with interbody fusion, which can be performed anteriorly (anterior lumbar interbody fusion/oblique lateral interbody fusion), especially if the disk height needs to be restored, or posteriorly (posterior lumbar interbody fusion/transforaminal lumbar interbody fusion). Unfortunately, there has not been enough conclusive literature to help surgeons choose the optimal approach [6,12].

Although the timing of surgery depends on the presence or progression of a neurological deficit and concomitant injuries, the deformity should ideally be stabilized as soon as possible to mobilize the patient, as reduction becomes more difficult with time [4,24].

In patients with deficits, the early surgical intervention consists of the extensive spinal canal and neuroforamina decompression and clearance of all neurotoxic materials (bone, soft tissue fragments, and disk material) to reduce the risk of neurologic injury during the reduction of the slipped vertebra [1,4,8].

Successful surgical treatment can be expected to improve neurologic symptoms, ambulation, and pain, except in patients with complete neurologic damage or nerve root transection. The timing of decompression and the extent of spinal canal narrowing are the most critical factors affecting neurologic recovery [24,25].

Besides neurological injury, surgical complications occurred in 22% of all documented cases, mainly due to implant failure in isolated posterior spinal fusion or nonoperative cases due to instability [6,8]. Therefore, the literature considers circumferential fusion (360°) with adequate decompression as the best procedure to ensure solid bony healing, especially in severe injuries with documented disk lesions. It thus leads to immediate restoration of physiological sagittal alignment of the spine, shorter rehabilitation times, and good results [1,3,4,8,18,21,22,24].

## Conclusions

Traumatic lumbosacral anterolisthesis is a rare, severe, and unstable three-column lesion. A thorough initial examination is required to avoid misdiagnosis and detect multiple associated injuries, particularly neurologic impairment. CT and MRI scans are valuable adjuncts to radiographs for evaluating bony and disk injuries, facilitating the selection of appropriate surgical procedures. The literature review indicates that decompression, reduction, and instrumentation with fusion are generally recommended. Circumferential fusion is the standard procedure when the disk is injured. In short- and medium-term follow-up, most of the described cases report satisfactory results. However, the long-term prognosis is still uncertain because of the small number of reported cases with heterogeneous outcomes and limited follow-up. Therefore, further sound studies are needed to guide spine surgeons in managing this entity.
